# Oxidative Stress, Hypoxia, and Autophagy in the Neovascular Processes of Age-Related Macular Degeneration

**DOI:** 10.1155/2014/768026

**Published:** 2014-02-23

**Authors:** Janusz Blasiak, Goran Petrovski, Zoltán Veréb, Andrea Facskó, Kai Kaarniranta

**Affiliations:** ^1^Faculty of Biology and Environmental Protection, Department of Molecular Genetics, University of Lodz, 90-236 Lodz, Poland; ^2^Department of Ophthalmology, Faculty of Medicine, University of Szeged, Szeged 6720, Hungary; ^3^Stem Cells and Eye Research Laboratory, Department of Biochemistry and Molecular Biology, Medical and Health Science Center and Apoptosis and Genomics Research Group of the Hungarian Academy of Sciences, University of Debrecen, Debrecen 4010, Hungary; ^4^Department of Ophthalmology, Institute of Clinical Medicine, University of Eastern Finland, 70211 Kuopio, Finland; ^5^Department of Ophthalmology, Kuopio University Hospital, 70211 Kuopio, Finland

## Abstract

Age-related macular degeneration (AMD) is the leading cause of severe and irreversible loss of vision in the elderly in developed countries. AMD is a complex chronic neurodegenerative disease associated with many environmental, lifestyle, and genetic factors. Oxidative stress and the production of reactive oxygen species (ROS) seem to play a pivotal role in AMD pathogenesis. It is known that the macula receives the highest blood flow of any tissue in the body when related to size, and anything that can reduce the rich blood supply can cause hypoxia, malfunction, or disease. Oxidative stress can affect both the lipid rich retinal outer segment structure and the light processing in the macula. The response to oxidative stress involves several cellular defense reactions, for example, increases in antioxidant production and proteolysis of damaged proteins. The imbalance between production of damaged cellular components and degradation leads to the accumulation of detrimental products, for example, intracellular lipofuscin and extracellular drusen. Autophagy is a central lysosomal clearance system that may play an important role in AMD development. There are many anatomical changes in retinal pigment epithelium (RPE), Bruch's membrane, and choriocapillaris in response to chronic oxidative stress, hypoxia, and disturbed autophagy and these are estimated to be crucial components in the pathology of neovascular processes in AMD.

## 1. Introduction

Environmental risk factors can interact with genetic factors or a sequence of disease-involving genes characteristic for each individual. These confounding factors can complicate the phenotype of the diseases and prevent identification of primary element(s) in different pathologies. Indeed, it is hard to determine whether any particular pathology is linked with the stress caused by a risk factor or if it is a consequence of the pathology or a combination of both. Oxidative stress is associated with many age-related diseases including age-related macular degeneration (AMD) [1]. Cells possess many different protective mechanisms to combat the acute detrimental effects of oxidative stress; however, chronically elevated stress can induce local pathological changes which may be irreversible. This can be due to a reduced cellular defense reaction towards the stress that subsequently leads to increased detrimental changes, and, eventually, a vicious cycle forms in which oxidative stress impairs the antioxidant properties of the cells and facilitates further the extent of stress. Damaged cellular components, which are no longer functional, should be degraded by cellular clearance systems including autophagy, which is a self-eating process triggered by oxidative stress and hypoxia. If the autophagic degradative pathway is faulty, an accumulation of damaged proteins as aggregated deposits takes place that may cause anatomical obstacles to physiological processes [1]. Therefore, the triplet of oxidative stress-hypoxia-impaired autophagy may play an important role in the pathophysiology of AMD. This review will elaborate the interplay between these triplet factors and how they associate with neovascular processes occurring in this disease.

## 2. Age-Related Macular Degeneration 

**Figure 1:**
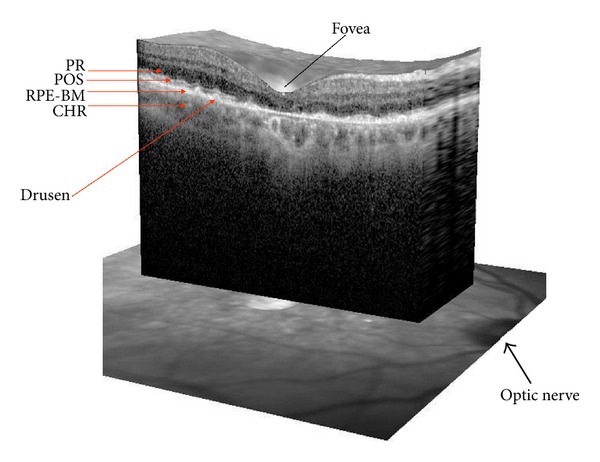
Optical coherence tomography (OCT) showing the major structures of the human retina in dry AMD patient. The structures marked are fovea, photoreceptors (PR), photoreceptor outer segments (POS), RPE-Bruch's complex (RPE-BM), and choroid (CHR). Drusen accumulate between RPE and Bruch's membrane. Optic nerve has been shown by thick arrow.

AMD is the main cause of irreversible vision loss in the elderly people accounting for about half of the newly registered cases of blindness in the developed world. The age-related increase in prevalence of the advanced form of the disease has been estimated to rise and reach an overall morbidity of 50% by the year 2020 [2, 3]. The initial symptoms of AMD include loss of central visual acuity, a subjective impression of the curvature of straight lines or metamorphopsia, and gradually enlarging central scotoma. The disease has a multifactorial etiology involving various environmental, demographic, and genetic risk factors. Although age is the most significant risk factor in AMD, the disease is also associated with hypertension, atherosclerosis, smoking, fat-rich diet, obesity, genetics, and epigenetics [4–7]. The abundance and complex interactions between all these risk factors limit the effectiveness of therapeutic options. The macular structures undergoing pathological changes are functionally and anatomically distinct but related, that is, photoreceptors, retinal pigment epithelium (RPE), Bruch's membrane, and choriocapillaris (Figure 1). The central processes involved in AMD pathology are, in the early or dry type of the disease, lipofuscinogenesis, autophagocytosis, and drusenogenesis, accompanied or followed by inflammation and neovascularization in the wet type of AMD (see later) [1].

## 3. Association between Pathophysiological Factors in AMD

Since the retina is one of the tissues with the most intense blood flow in the organism, retinal structural changes associated with AMD can result in disturbance of retinal blood flow. The Blue Mountains Eye Study indicated that AMD is associated with focal arterioral narrowing and arteriovenous nicking in the retinal vasculature [3]. Vascular factors have been reported to play an important role in the AMD pathogenesis. There is a growing body of evidence that choroidal and retinal blood flow are diminished in AMD [8, 9]. Therefore, hypoxia is thought to be associated with the progression of AMD. Normal retinal physiology and all forms of AMD are regulated by vascular growth factors, but under certain conditions, the physiological balance shifts to pathological [10]. Vascular dysfunctions may result in oxidative stress, that is, overproduction of ROS, which induces further changes in the retinal vessels. Such changes can also be evoked by hypoxia, since it stimulates synthesis and release of hypoxia-inducible factor-1 (HIF-1) and vascular endothelial growth factor (VEGF) that contribute to neovascularization (NV). Noteworthy here is that hypoxia is a classical inducer of autophagy [11], which in turn can be stimulated by oxidative stress in an attempt to clear macromolecules damaged by ROS. Therefore, interaction between hypoxia, oxidative stress, and autophagy exists and it can be postulated to play an important role in the pathogenesis of AMD (Figure 1).

## 4. Oxidative Stress in AMD

Oxidative stress results from disturbances in the prooxidative/antioxidative cellular balance due to elevated levels of oxidation reactions producing ROS: superoxide anion (O_2_^−∙^), hydroxyl radical (OH^∙^), hydrogen peroxide (H_2_O_2_), and singlet oxygen (1O_2_). ROS can be formed in many ways, (1) as a product of the respiratory chain in mitochondria, photochemical and enzymatic reactions, (2) as a result of the exposure to UV light, (3) ionizing radiation, or (4) heavy metal ions. Hydrogen peroxide is a molecule with low reactivity, but it can readily penetrate cell membranes and generate the most reactive form of oxygen—the hydroxyl radical, via the Fenton reaction: H_2_O_2_ + Fe^2+^ → Fe^3+^ + OH^−^ + OH^∙^.

ROS play an important role in the regulation of many physiological processes involved in intracellular signaling [12], but they can also induce serious damage to biomolecules. Lipid peroxidation (autooxidation) is a process of oxidation of polyunsaturated fatty acids due to the presence of several double bonds in their structure and it involves production of peroxides and reactive organic free radicals. The latter can then react with other fatty acids, initiating a free radical reaction cascade. ROS also attack structural and enzymatic proteins by the oxidation of residual amino acids, prosthetic groups, formation of cross links, and protein aggregates as well as proteolysis. The inactivation of key proteins can have serious consequences in the vital metabolic pathways. ROS can also react with nucleic acids attacking the nitrogenous bases and the sugar phosphate backbone. Furthermore, they can evoke single- and double-stranded DNA breaks. The cells' inability to repair the incurred damage may lead to death or, alternatively, mutations may occur in the DNA leading to carcinogenesis or development of neurodegenerative diseases [13].

Mitochondrial DNA (mtDNA) is more susceptible to oxidative damage than its nuclear counterpart [14]. Mutations in mtDNA can cause disturbances in the respiratory chain and loss of control of ROS production. mtDNA is not protected by histones or other associated proteins, and since it has intronless regions and a high transcription rate, it has a higher susceptibility to oxidative modifications in its coding region. The much less effective repair system for mtDNA damage may be the cause for accumulating oxidative stress and its consequences thereof.

Oxidative damage of the cellular components plays an important role in the senescence process [15]. The amount of accumulated damage increases with age due to impairs in the DNA repair system and an intensified oxidative stress and decreased antioxidant defense [14]. Mutations in the key DNA repair genes result in an impaired recognition system and an inefficient repair of DNA damage, which accelerates the aging of the organism, leading to age-related disruptions in cellular and tissue functions. The aging of the organism is inevitable since the formation of ROS is a result of normal daily cellular metabolism. Therefore, cells have developed complex defense mechanisms to combat both the formation of ROS and the impacts of their actions.

Cells which are the most sensitive to oxidative damage are nonproliferative postmitotic cells, including photoreceptors and RPE cells, since they do not possess any DNA damage detection systems in the cell cycle checkpoints. Furthermore, the macular environment promotes the production of ROS. In the macula, the predominant photoreceptors are cones, which have higher demand and production of energy than rods and, therefore, higher demand for oxygen [16–19]. In addition, rods and cones differ in their susceptibility to oxidative stress, with cones exhibiting a higher sensitivity to free radicals [20]. The macula is constantly exposed to a high metabolic rate and oxidative stress due to the high partial pressure of choriocapillaris and the amount of polyunsaturated fatty acids (PUFAs) from the retinal outer segments [21]. Oxidated PUFAs are not efficiently digested in the lysosomes of aged RPE cells and become deposited in the form of lipofuscin (Figure 1). This is thought to be important in inducing the formation of drusen between the RPE cells and Bruch's membrane. Lipofuscin is a chromophore, serving as the main RPE photosensitizer [22], which, after absorbing a high-energy photon, especially that of blue light, undergoes a variety of photochemical reactions involving ROS formation, which, in turn, evoke photochemical damage in the retina and RPE cells [23].

## 5. Hypoxia in AMD Pathophysiology

In the majority of human tissues, the concentration of oxygen ranges from 3 to 5% and a decline below this range is usually considered hypoxia [24]. In a more general sense, hypoxia may be defined as a decrease in available oxygen reaching the tissues of the body. Hypoxia should not be considered dangerous in itself, because many human beings live at high altitudes and they have adapted to live under reduced oxygen tension. However, some serious consequences of hypoxia can occur, including failure of energy balance causing ATP depletion, ROS-induced damage of cellular components, uncontrolled excitatory neurotransmitter release, inflammation and stimulation of the immune system, and delayed cell death [25]. The cellular reactions to hypoxia evoke change in expression of many genes. HIF-1 is the main regulator of oxygen homeostasis and consists of HIF-1*α* and HIF-1*β* subunits [26]. HIF-1 regulates the expression of hundreds of genes to ensure cell survival under conditions of hypoxic stress, aimed at restoring O_2_ homeostasis. Under hypoxic conditions, HIF-1 interacts with pyruvate dehydrogenase kinase 1 (PDK1), lactate dehydrogenase A (LDHA), and BNIP3 and BNIP3L proteins involved in the mitochondrial autophagy pathway [26–30].

In general the inner retina layers are better protected from ischemic stress than other parts of the central nervous system; these cells are capable of recovering after an acute hypoxic insult, but not after chronic retinal ischemia and hypoxia which can lead to cell death and irreversible visual impairment [31–34]. There is a report that retinal blood flow is disturbed in both dry and wet or neovascular type of AMD [35]. Moreover, the reduction in choroidal perfusion has been positively correlated with AMD progression [36]. These observations indicate that hypoxia is associated with AMD. Nonetheless, the question remains: is the hypoxia a consequence or a reason for the disease? The answer to this question is hard to formulate as long as our knowledge about very early mechanisms of AMD is so scarce. Measurement of oxygen tension, perfusion pressure, and blood flow rate indicates that hypoxia is the result of diminished choroidal blood circulation [34, 35].

Inflammation and local hypoxia are present in the aging of choriocapillaris, RPE cells, and neural retina [36]. During inflammation, hypoxia in the retinal cells may result from increased consumption of oxygen due to the increased metabolic activity of the inflamed retina. In general, AMD can be characterized as presence of chronic inflammatory state, with local infiltration of inflammatory cells, higher circulatory levels of proinflammatory cytokines, and complement components [37]. Although the precise mechanisms underlying such chronic inflammation are not yet known, it can be speculated that oxidative stress-related damage to macromolecules in the retina may activate redox-regulated transcription factors, such as nuclear factor kappa B (NF-*κ*B), and increase the expression of proinflammatory molecules. NF-*κ*B can also be activated by advanced glycation end products (AGEs) and their receptor (RAGE) that are all overexpressed in AMD [38, 39]. Chronic oxidative stress and the presence of chronic inflammation decrease the ability of RPE cells to remove damaged or nonfunctional proteins via the lysosomal clearance system, including autophagy (Figure 1) [1].

## 6. Autophagy: A Cellular Clearance System in AMD 

**Figure 2:**
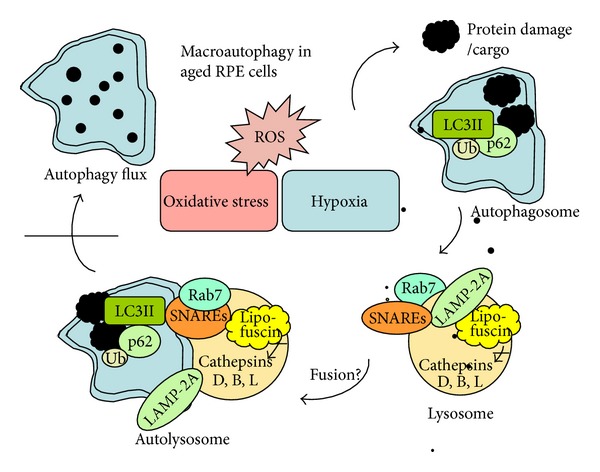
Schematic presentation of the macroautophagy process in aged retinal pigment epithelial (RPE) cells. Oxidative stress, ROS, and hypoxia lead to protein damages and aggregation that induces autophagy. The substrate (cargo) for autophagy is degraded by lysosomal acid hydrolases, including cathepsins D, B, and L, after the fusion of lysosome and autophagosome that form autolysosome. Rab7, LAMP-2A, and SNAREs proteins are critical for the lysosome and autophagosome fusion process. Ubiquitin (Ub), LC3II, and p62 are complexed to the cargo and connect autophagy to the proteasomal clearance system. Macroautophagy is prevented in AMD, since lysosomal lipofuscin disturbs cathepsin activity and autophagy flux. Fusion mechanisms in the RPE cells are under investigation.

Autophagy, also known as self-eating, is a process of degradation and elimination of no longer needed intracellular components [40]. It contributes to the equilibrium between the production of proteins and organelles and their clearance. In addition to removing defective structures, autophagy is a means of providing macromolecules for energy generation under conditions of nutritional starvation [41]. Autophagy can be categorized into at least three classes: macroautophagy, chaperone-mediated autophagy (CMA), and microautophagy. A marked reduction of macroautophagic activity with aging has been associated with an increase in CMA [42], while microautophagy has been less described in the pathogenesis of AMD. The main focus here will be on macroautophagy (often referred to as just autophagy), which involves the formation of autophagosomes, double-membrane vesicles, in a multistep process. The autophagosomes combine with lysosomes and degrade their contents with several acidic hydrolases. This process is mediated by more than 30 autophagy-related (Atg) proteins. Macroautophagy consists of two subsets: autophagy of specific organelles and selective macroautophagy. Although substantial progress has been made in understanding the complex mechanisms regulating autophagy, many interactions involved in controlling this process have not been adequately described so far [40]. ROS inhibits the activity of the signaling protein mammalian target of rapamycin (mTOR) that evokes dephosphorylation of Atg13, activation of Serine/threonine-protein kinases-ULKs, and recruitment of FIP200. The ULKs-Atg13-FIP200 complex plays a crucial role in the formation of double-membrane autophagic vacuoles—the autophagosomes [43–45]. Other important proteins in the autophagosome formation are Atg14L (Atg14L-Beclin 1-hVps34-p150) and UVRAG (UVRAG-Beclin 1-hVps34-p150) complexes. Atg8/LC3 is a ubiquitin-like protein which connects autophagy to the proteasomal clearance system via ubiquitin and p62 binding sites (Figure 2) [1, 46, 47]. LC3 is formed by the cleavage of its precursor by Atg4 and modification with phosphatidylethanolamine (PE) mediated by Atg7, Atg3, and Atg16L complex. Atg7 promotes the formation of the Atg10–Atg12, Atg5–Atg12, and LC3-PE conjugates, which also play an essential role in the creation of the final form of autophagosome [43–45]. Atg7, Atg3, and Atg10 are sensitive to redox signaling, which supports the association between autophagy and oxidative stress. We recently described that the SQSTM1/p62 protein is a connecting link between autophagy and proteasome-mediated proteolysis, which is upregulated under exposure to various oxidative stimuli in AMD donor samples [48]. One critical step in autophagy clearance is fusion of autophagosome and lysosome that is regulated by Rab7, LAMP-2, and a large superfamily of specific small proteins, SNAREs (SNAP (soluble N-ethyl-maleimide-sensitive-fusion-protein attachment protein) receptors) (Figure 2) [49]. SNAREs increase the permeability of membranes and drive the actual membrane opening leading to fusion of the contents of the two organelles [50]. After fusion, the lysosomal proteases, which include cathepsins D, B, and L, degrade the sealed cargo proteins [51, 52]. The enzyme activity of the cathepsins is suppressed by oxidized lipoprotein, emphasizing the mutual relationship between oxidative stress and autophagy [53]. Impaired lysosomal function evokes decreased autophagy flux that may be a critical aspect of RPE cell degeneration and AMD development [54].

## 7. Neovascularization in AMD

**Figure 3:**
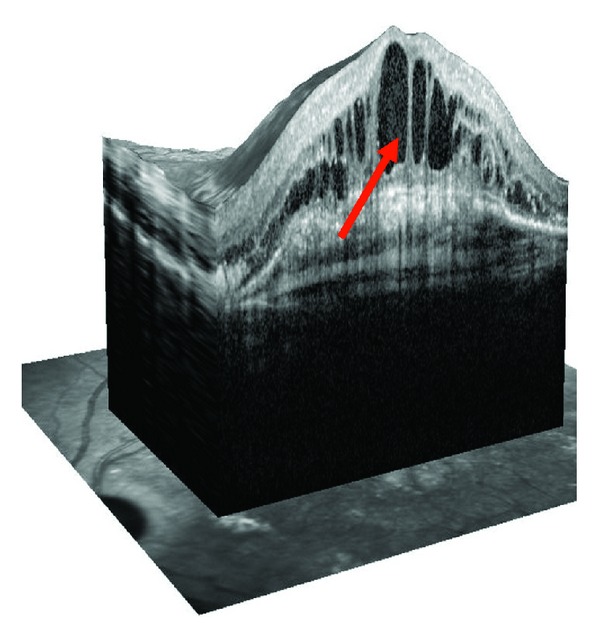
Optical coherence tomography (OCT) showing the edema of the human retina in wet AMD patient (arrow).

Unlike dry type of AMD, wet or exudative AMD is hallmarked by defects in Bruch's membrane or the outer blood-retina barrier, as well as formation of choroidal neovascularizations (CNVs) and/or subretinal neovascular membranes (SRNVMs). CNV evokes retinal edema that is one diagnostic criterion for wet AMD (Figure 3). Besides these, a direct pathological contact between RPE and endothelial cells (ECs) can enhance the proangiogenic potential of the ECs to proliferate and migrate, similar to the process induced by hypoxia. The linkage between pathological changes in the RPE and the development of CNVs is the result of a multifactorial interplay of oxidative stress, hypoxia, and autophagy in wet AMD pathogenesis.

**Figure 4:**
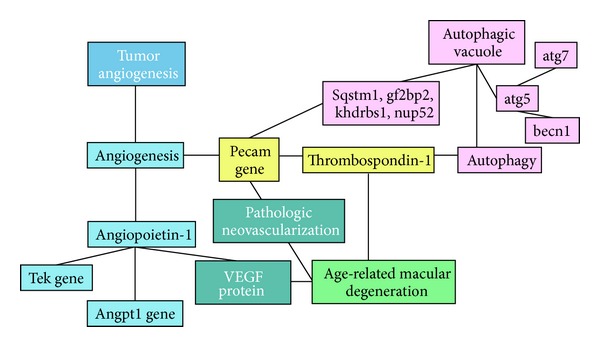
EpiphaNet interactive map for angiogenesis, autophagy, and age-related macular degeneration (AMD) (reproduced from the EpiphaNet software for better visibility of interactions; Angpt1 and Tek are genes encoding angiopoietin-1 and its receptor, respectively; Pecam gene encodes platelet endothelial cell adhesion molecule (PECAM-1); Igf2 bp2: insulin-like growth factor 2 mRNA-binding protein 2; Khdrbs1: KH domain containing, RNA binding, signal transduction associated 1; Nup52: nucleoporin 52, component of the p62/sequestome 1 complex; Sqstm1: sequestome 1; VEGF: vascular endothelial growth factor).

Using EpiphaNet' interactive tool software (http://epiphanet.uth.tmc.edu/) and a mesh of linkage terms such as “angiogenesis,” “autophagy,” and “age-related macular degeneration,” one can find putative connector pathways for these processes (Figure 4). Platelet endothelial cell adhesion molecule (PECAM-1) and Thrombospondin 1 (TSP-1) stood out as such linkage molecules in the EpiphaNet analysis, and, indeed, they have been shown in the literature to have a mutually reciprocal relationship with angiogenesis [55]. Impaired expression of TSP-1 in rodent eyes with AMD has been previously described, and the decreased TSP-1 level in Bruch's membrane and choroidal vessels during AMD has been claimed as the possible cause of CNV [56]. Similarly, interaction analysis between “autophagy” and “angiogenesis” found TSP-1 as a common denominator between angiogenesis, autophagy, and AMD, having been detected as antiangiogenic molecule and a target for antineovascular therapy as well [57]. Besides these, Tek and Angpt1 gene-encoded angiopoietin-1 and receptor, respectively, connect the physiologic and pathologic neovascularization processes with AMD. Indeed, Angp1 has been detected in surgically excised CNV membranes obtained surgically from eyes with AMD, while Tie2/Tek immunoreactivity has been observed in the vascular structures of CNVs, as well as the RPE monolayer [58].

## 8. Concluding Remarks

There is a growing body of evidence suggesting that oxidative stress is linked to hypoxia in AMD. Similar relationships probably also exist between oxidative stress and deficient autophagy as well as between faulty autophagy and hypoxia in AMD. Therefore, the trio of oxidative stress-hypoxia autophagy may represent a self-fueling chain reaction of events, which accelerates AMD progression. However, the question “Who was the first?” still remains open. In many cases, oxidative stress is a primary source of ROS, which can damage cellular biomolecules and organelles. Their accumulation should lead to their degradation by cellular clearance systems of which autophagy is one of the most important. However, since autophagy can be disturbed by the activities of ROS, this may lead to detrimental protein accumulation. These negative properties of ROS may also affect vascular molecules, disturbing the choroidal blood flow and inducing hypoxia. The relationship between hypoxia and ROS production has not been firmly established but many of the ambiguous results in this topic can be attributed to the lack of adequate techniques to detect or determine the exact ROS levels in the retina. Therefore, further studies investigating the mechanisms underlying the mutual associations between oxidative stress, hypoxia, and autophagy in the different forms of AMD, in particular neovascular AMD, should aim at clarifying this issue.
